# Opposing effects of final population density and stress on *Escherichia coli* mutation rate

**DOI:** 10.1038/s41396-018-0237-3

**Published:** 2018-08-07

**Authors:** Rok Krašovec, Huw Richards, Danna R. Gifford, Roman V. Belavkin, Alastair Channon, Elizabeth Aston, Andrew J. McBain, Christopher G. Knight

**Affiliations:** 1 Faculty of Biology, Medicine and Health, The University of Manchester, Manchester, M13 9PT UK; 2 Faculty of Science and Engineering, The University of Manchester, Manchester, M13 9PT UK; 3 School of Engineering and Information Sciences, Middlesex University, London, NW4 4BT UK; 4 School of Computing and Mathematics, Keele University, Keele, ST5 5BG UK

## Abstract

Evolution depends on mutations. For an individual genotype, the rate at which mutations arise is known to increase with various stressors (stress-induced mutagenesis—SIM) and decrease at high final population density (density-associated mutation-rate plasticity—DAMP). We hypothesised that these two forms of mutation-rate plasticity would have opposing effects across a nutrient gradient. Here we test this hypothesis, culturing *Escherichia coli* in increasingly rich media. We distinguish an increase in mutation rate with added nutrients through SIM (dependent on error-prone polymerases Pol IV and Pol V) and an opposing effect of DAMP (dependent on MutT, which removes oxidised G nucleotides). The combination of DAMP and SIM results in a mutation rate minimum at intermediate nutrient levels (which can support 7 × 10^8^ cells ml^−1^). These findings demonstrate a strikingly close and nuanced relationship of ecological factors—stress and population density—with mutation, the fuel of all evolution.

## Introduction

How and why the rate of spontaneous genetic mutation varies is a fundamental and enduring biological issue [1]. Mutation rate can vary both among species [2] and within a genotype [3]. Intra-genotypic variation can depend upon stressful environmental conditions, such as nutrient limitation, growth-rate reduction, high osmotic pressure, low pH, extreme shifts in temperature or various DNA-damaging agents [4]. In these environments, double-stranded breaks can induce stress responses that in turn increase mutation rates via DNA polymerases with different error rates [5]—a phenomenon known as stress-induced mutagenesis (SIM).

Recently, we found that, across microbes, the mutation rate of a particular genotype is closely associated with the final density to which the population grows (*D,* i.e. the carrying capacity of the environment divided by its volume) [6]. In this so-called density-associated mutation-rate plasticity (DAMP), bacterial and yeast populations show a power law (log–log linear) reduction in mutation rate with *D* when grown in a defined minimal medium with glucose as the sole carbon source [6, 7]. DAMP and SIM modify mutation rates in *Escherichia coli* via different genetic pathways. DAMP requires a Nudix hydrolase protein, whose primary role is degrading highly mutagenic 8-oxo-dGTP [8], while error-prone polymerase Pol IV is not involved in DAMP [6]. Differences in the underlying mechanism and the fact that the densest populations, experiencing the highest stress, show the lowest mutation rates, suggest that DAMP is not obviously associated with stress.

Growth in minimal medium on a single carbon source does not, however, reflect the environmental complexity or range of population densities experienced by many species. *E. coli* population density in host environments varies over five orders of magnitude among host species, and can be higher than 10^9^ colony-forming units per gram of faeces (reviewed in ref. [9]). As the highest population densities, with the greatest competition, rely on high nutrient availability, we reasoned that the addition of nutrients to minimal nutrient environments could indirectly increase both population densities and the level of stress. We therefore hypothesised that the effects of density and stress on mutation rates, DAMP and SIM respectively, will act in opposition to one another across such a nutrient gradient—DAMP decreasing mutation rate and SIM increasing it as nutrients and final population density increase.

**Fig. 1:**
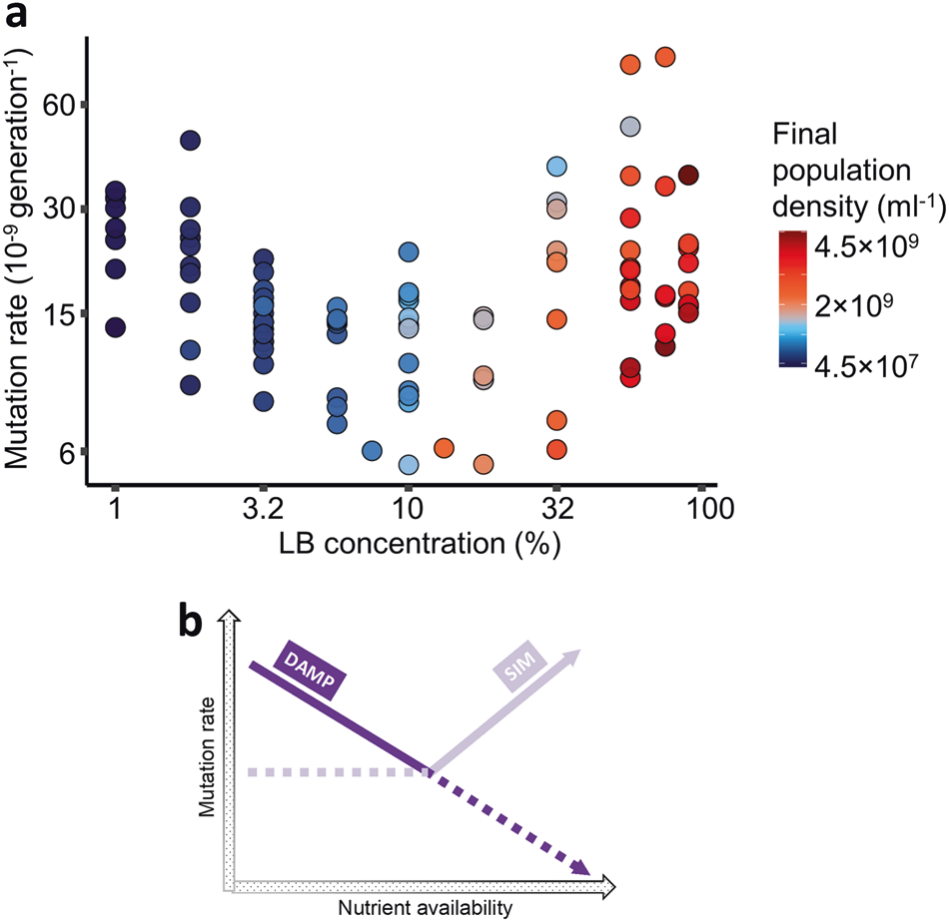
Effect of nutrient availability on mutation rate **a**) to rifampicin resistance in wild-type *E. coli* K-12 MG1655 (*N* = 97). Cells were grown in Davis minimal medium mixed with 1–90% of lysogeny broth (LB) medium. Colours represent final population density measured by colony-forming units (see Fig. S2 for details). See Figure S4 for a plot of mutation rate directly against final population density (measured by ATP-based assay) and S10 for mutation rates co-estimated with the relative fitness of resistant mutants and model S-I in Supplementary Information for analysis. Note the nonlinear axes. **b** Schematic of the mechanisms involved in mutation-rate plasticity (density-associated mutation-rate plasticity, DAMP, and stress-induced mutagenesis, SIM). At low nutrient availabilities, DAMP is present and SIM is absent. At higher nutrient availabilities, SIM becomes dominant. Solid lines correspond to the mutation-rate plasticity measured in **a**. Genetically removing one or the other mechanism (Figs. 2 and 3) reveals mutation-rate plasticity indicated by the dotted lines

Here we test this hypothesis by determining *E. coli* mutation rates across a nutrient gradient, while genetically manipulating DAMP and SIM independently. As hypothesised, we identify genetically separable and opposed associations of mutation rate with nutrient availability—a negative association requiring *mutT* (DAMP) and a positive association requiring polymerases IV and V (*dinB* and *umuC*, respectively; SIM). We find that these associations combine to minimise average mutation rates in environments with intermediate nutrient availability and final population density (Fig. 1b).

## Results

We assayed mutation rates to rifampicin resistance using fluctuation tests in *E. coli* K-12 MG1655 grown across a gradient of nutrient availability: a range of concentrations (1–90% vol/vol) of lysogeny broth (LB) mixed with Davis minimal (DM) medium (LB/DM). We find that the relationship of mutation rate to LB concentration is non-linear (Fig. 1a, likelihood ratio test of a quadratic effect of log nutrient availability on log mutation rate: *N* = 97, LR_8,7_ = 105, *P* = 1.2 × 10^−24^, model S-I in Supplementary Information). Repeating this experiment using a different marker of mutation (nalidixic acid resistance) gives a similar nonlinear relationship (Fig. S1). Mutation rate to both rifampicin and nalidixic acid resistance decreases as LB/DM is increased from 1 to 10% LB (increasing final population density, *D*, from 4.5 × 10^7^ to 1 × 10^9^ cells ml^−1^, Figs. S2–S5). This is comparable to DAMP in DM with glucose [6, 7]. However, mutation rate increases again in richer media, with 90% LB reaching similar or higher mutation rates than in 1% LB.

**Fig. 2:**
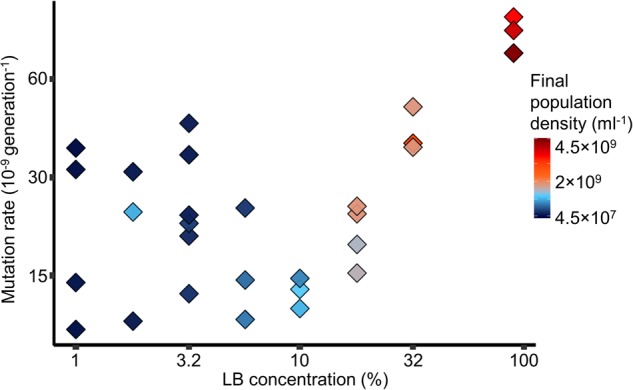
Effect of nutrient availability on mutation rate to nalidixic acid resistance in cells without DAMP (Δ*mutT*, *N* = 30). Cells were grown in Davis minimal medium mixed with 1–90% of lysogeny broth (LB) medium. Colours represent the final population density measured by colony-forming units (see Fig. S2 for details). See Fig. S6 for a plot of mutation rate directly against final population density and S11 for mutation rates co-estimated with the relative fitness of resistant mutants. Nalidixic acid resistance is used as the marker here rather than rifampicin resistance (as in Fig. 1) because the mutation rate of this Δ*mutT* strain is too high to assay with rifampicin (for which at least 69 different resistance mutations are known [13], the ‘target size’ for nalidixic acid resistance is much smaller). Directly comparable data for wild-type MG1655 mutation rate to nalidixic acid resistance are shown in Fig. S1. Note the nonlinear axes

We next asked whether the increase in mutation rate at higher nutrient availability is genetically separable from the decrease in mutation rate due to DAMP. DAMP in *E. coli* requires the 8-oxo-dGTP diphosphatase MutT protein, meaning that, in minimal medium with glucose, the mutation rate in a Δ*mutT* mutant does not decrease with increased nutrient concentration [6]. We therefore performed fluctuation tests to nalidixic acid resistance in LB/DM with a Δ*mutT* mutant. We find that in LB/DM, as in DM with glucose, mutation rate in Δ*mutT* shows no relationship with increased nutrients or final population density below 10% LB (Fig. 2). However, even more clearly than in the wild types (both MG1655 (Fig. 1a) and the immediate parent of the Δ*mutT* mutant (Fig. S7)), mutation rate of the Δ*mutT* mutant increases with the nutrient availability above 10% LB (final population density of ~1 × 10^9^ cells ml^−1^). The only other *E. coli* mutant reported not to exhibit DAMP is *E. coli* K-12 MG1655 Δ*luxS* [7]. However, the deficiency in DAMP of this mutant is functionally complemented by added aspartate [7], and LB is a medium rich in amino acids [10]. If variation in mutation rate at 10% LB/DM and below is the same phenomenon as DAMP, we expect this mutant strain to behave more similarly to a wild-type than the Δ*mutT* mutant. We find that the Δ*luxS* mutant’s mutation rate is indistinguishable from the wild-type MG1655 across LB/DM environments (Fig. S7, *N* = 167, likelihood ratio tests of the *luxS* deletion on the interaction between the quadratic response of mutation rate to LB concentration and genotype [*N* = 167, LR_11,12_ = 2, *P* = 0.16], and on the fixed effect of genotype [*N* = 167, LR_11,10_ = 1.4, *P* = 0.24]).

**Fig. 3:**
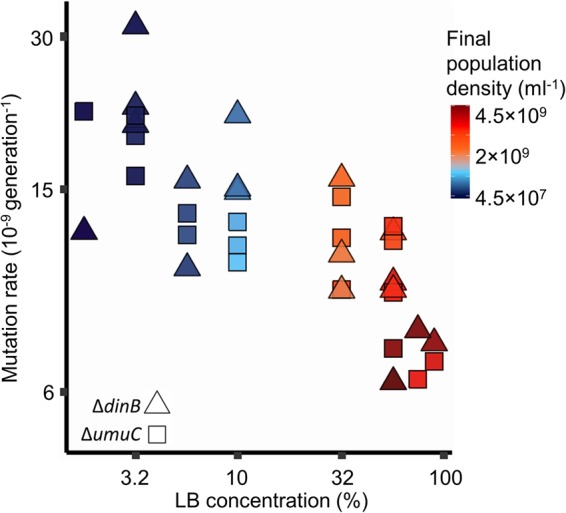
Effect of nutrient availability on mutation rates to rifampicin resistance in cells without error-prone polymerases Pol IV (Δ*dinB*, *N* = 18, triangles) and Pol V (Δ*umuC*, *N* = 18, squares). Cells were grown in Davis minimal medium mixed with 1–90% of lysogeny broth (LB) medium. The mutation rates of the two strains are not distinguishable (likelihood ratio test of the effect of genotype [*N* = 36, LR_9,8_ = 2.6, *P* = 0.11]). There is no evidence of a nonlinear relationship of log mutation rate with nutrient availability (likelihood ratio test of a quadratic effect [*N* = 36, LR_8,7_ = 0.15, *P* = 0.70]), but there is a highly significant linear effect of nutrient availability (likelihood ratio test of a linear effect [*N* = 36, LR_7,6_ = 45, *P* = 1.6 × 10^−11^]), see model S-III in Supplementary Information. Colours represent final population density measured by colony-forming units (see Fig. S2 for details). See Figure S8 for an equivalent plot using final population density and S12 for mutation rates co-estimated with the relative fitness of resistant mutants. Note the nonlinear axes

The fact that mutation rate increases at high LB concentrations in a Δ*mutT* mutant (Fig. 2), where DAMP is absent, suggests that high LB concentrations increase the mutation rate via a DAMP-independent mechanism. We hypothesised that higher LB concentrations increase the level of stress (e.g. by promoting competition), thereby causing error-prone polymerases Pol IV and Pol V (coded by *dinB* and *umuCD*, respectively) to increase the mutation rate at very high LB concentrations. We tested this hypothesis by estimating mutation rates to rifampicin resistance in *E. coli* Δ*dinB* and Δ*umuC* mutants growing in LB/DM. We find that, unlike *E. coli* MG1655 (Fig. 1a) and Δ*mutT* (Fig. 2), mutation rates of the Δ*dinB* and Δ*umuC* deletants (Fig. 3, Model S-III in Supplementary Information) decrease with increasing nutrients above 10% LB (above final population densities of 7 × 10^8^ cells ml^−1^, Fig. S8). This continued decrease indicates that these polymerases are required for the rise in mutation rates as nutrients increase, and that DAMP continues to affect mutation rates at high nutrient levels.

The fitness effects of resistance mutations are known to be variable among nonselective environments, particularly for rifampicin [11]. This variation has the potential to give artefactual differences in mutation rates among environments. We therefore estimated the fitness effects of resistance mutations in the fluctuation tests reported in Figs. 1–3 (Fig. S9). As expected, resistance mutations were, on average, somewhat deleterious across nutrient environments for both rifamipcin and nalidixic acid. Surprisingly, the average effect was least deleterious in intermediate nutrient environments. This suggests that, if anything, mutation rates in intermediate nutrient environments are over-estimated, relative to high and low nutrient environments. Thus, the results reported in Figs. 1–3 are robust to environmentally-dependent fitness effects of resistance mutations (Fig. S10–12).

## Discussion

**Fig. 4:**
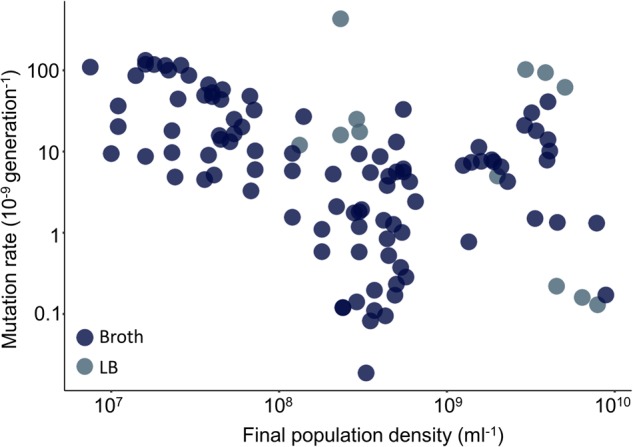
Distribution of *E. coli* mutation rates estimated in rich media and published from 1943 to 2016 (re-plotted from ref. [6], *N* = 111). Broth—rich nutrient medium, LB—rich lysogeny broth medium. These data also include a range of genotypes and phenotypic markers (Supplementary Data File). Note the nonlinear axes

Our previous work on DAMP [6], contained a paradox. In the laboratory, *E. coli* displays a substantial and highly significant decrease in mutation rate associated with final population density, much more so than the related bacterium *Pseudomonas aeruginosa* PAO1. Yet, in the published literature, of all 26 microbial species with appropriate data (including *P. aeruginosa*), the one with least negative association was *E. coli*. Here we have resolved that paradox. We have shown how two mechanistically independent plastic processes act on the mutation rate: DAMP—apparent at lower final population densities (<7 × 10^8^ cells ml^−1^)—causing mutation rate to *decrease* with increasing nutrient concentration; SIM—apparent at higher final population densities (>1 × 10^9^ cells ml^−1^)—causing mutation rate to increase with nutrient concentration. *E. coli* is the organism whose mutation rate has, across the last 75 years of literature, been measured across the broadest range of final population densities (7.5 × 10^6^–8.9 × 10^9^ cells ml^−1^) [6]. This means that, like Fig. 1a or Fig. S4, the published literature includes a range of rich media and shows a minimum in *E. coli*’s mutation rate at around 7 × 10^8^ cells ml^−1^ (Fig. 4). This explains why attempting to fit a linear trend to these data does not yield a steep negative relationship [6]. It is also consistent with DAMP acting at low final population densities and SIM at high densities across diverse published studies (111 individual estimates across 12 studies), as we find here in a single, controlled study. We cannot currently say what particular aspect(s) or component(s) of the rich medium used here is/are most important in effecting the observed mutation rate changes. However, the fact that a similar pattern is seen in published data using different ‘broth’ media suggests that, like DAMP itself [6], the interplay of opposing pressures on mutation rate that we have dissected here (Fig. 1b) is not unique to the particular media we used.

It is perhaps surprising that both error-prone polymerases Pol IV and Pol V are required for the increase in mutation rate observed here at high LB concentrations (Fig. 3), since, in some cases, only pol IV is required for SIM [12]. Nonetheless, in SIM, increases of base substitutions (as opposed to insertion or deletion events), which are the primary form of mutation underlying the rifampicin resistance assayed here [13], do frequently depend on both of these polymerases [14–16]. Such different molecular processes leave different signatures in the precise spectrum of mutations observed. However, the dependence of such mutational spectra on nutrient environment has recently been shown to be very complex [3]. It is thus unlikely that such spectra will explain the mechanisms of DAMP, SIM and potentially other environmental plasticity in mutation rates, which vary simultaneously, via different molecular mechanisms, even across a simple nutrient gradient. However, the sort of genetic separation of environmental effects reported here may in future help understand the complexities of those spectra, which have the potential to affect the course of evolution [17].

It seems likely a priori that the dynamics of population growth and cell division, which differ among nutrient environments, are involved in the mutation rate changes observed here. For instance, environmental differences that affect growth rate will in turn affect ploidy [18], which can affect mutation rate estimates [19]. Therefore, we cannot exclude the possibility that the effects of either DAMP or SIM on mutation rate considered here are mediated by some aspect(s) of the culture cycle that differ across different nutrient environments. Such dynamics are largely inaccessible to fluctuation tests, as used here, or indeed other standard methods of assaying mutation rate [20] that consider at least one full population growth cycle. Continuous culture, specifically continuous culture at a fixed population density, a turbidostat [21], may be a useful tool with which to factor out some of these dynamics. However, still better may be to examine the mechanistic detail of such dynamics directly using single-cell mutation monitoring approaches [22, 23].

Variation in mutation rate among members of a population can itself provide evolutionary advantages [24], and modulating the mutation rate in response to the environment could hypothetically allow organisms to optimise their rate of adaptation [25]. Such ‘optimal’ variation involves minimising mutation rates at high fitness, but allowing them to increase away from fitness peaks. However, environmental cues do not give direct information about an individual’s fitness. An individual simply receives information about the levels of particular molecules in the environment. These could give information, for instance, about the availability of a particular nutrient or about population density, and that information may be linked to mechanisms involved in the plastic control of mutation rate, e.g. Krašovec et al. [7]. But a cue indicative of high population density could be an indicator of an individual having high fitness (if it is part of a successful clone), or high competition and therefore low individual fitness. Similarly, unutilised nutrients may be indicative of a benign environment and therefore high fitness, or of a clone that has been unable to utilise resources and therefore low individual fitness. Organisms receive many environmental cues and may therefore be able to parse them to give a clear picture of the competitive environment, enabling appropriate responses [26]. How far this occurs in terms of mutation rate is unclear. And adaptive explanations are probably unnecessary to explain the existence of SIM and perhaps DAMP, given more direct and/or non-adaptive explanations [1, 27]. Nonetheless, it is reasonable to speculate about the evolutionary effects of these plastic mutation rate traits, whatever their origins, either evolutionarily or mechanistically in terms of environmental cues. The effect of minimising mutation rate in intermediate nutrient (and final population density) environments (Figs. 1a, S1 and S4) may be to minimise evolutionary change for organisms that are doing well in a relatively benign nutrient environment, but without excessive competition, which is potentially advantageous [25].

Without clearer evidence around the evolution of these traits and their effects on evolution beyond fluctuation tests, any reasoning about their adaptive effects remains speculative. Nonetheless, final population density and nutrient availability are focal points of microbial ecological competition [28, 29]. Microbes have evolved numerous strategies to sense and increase the acquisition of resources [30], and they possess efficient ways of sensing population density [31]. A threshold population density (known as quorum) is often required to regulate a diverse array of physiological activities [32], many of which promote stress tolerance [33]. SIM is not known to depend on quorum-sensing. Although DAMP requires LuxS, central to autoinducer 2 quorum sensing, in minimal media [7], we do not find any role for LuxS in the conditions studied here (Fig. S7). This is consistent with a metabolic rather than a quorum-sensing effect on DAMP [7]. Nonetheless, mutation rate can respond to the *luxS* genotype of a co-cultured strain [7], indicating that, in addition to the nutrient environment studied here, biotic environment also has a role in determining an organism’s mutation rate. The fact that mutation rate responds in such complex ways to these diverse environmental factors indicates that, for the de novo evolution of traits such as antibiotic resistance, ecological circumstances and evolutionary outcomes are tightly linked.

## Materials and methods

### Strains used in this study

*E. coli* K-12 strain KX1228 (Δ*luxS*) was derived from the wild-type K-12 MG1655 (*luxS*^+^) [31]. *E. coli* Δ*mutT* mutant is part of Keio collection [34] designated as JW0097-1 (F-, Δ*(araD-araB)567*, *ΔlacZ4787*(::rrnB-3), λ-, Δ*mutT790*::kan, *rph-1*, Δ*(rhaD-rhaB)568*, *hsdR514*). *E. coli* Δ*dinB* mutant is part of Keio collection designated as JW0221-1 (F-, Δ*(araD-araB)567*, *ΔlacZ4787*(::rrnB-3), λ-, Δ*dinB749*::kan, *rph-1*, Δ*(rhaD-rhaB)568*, *hsdR514*). *E. coli* Δ*umuC* mutant is part of Keio collection designated as JW1173-1 (F-, Δ*(araD-araB)567*, *ΔlacZ4787*(::rrnB-3), λ-, Δ*umuC773*::kan, *rph-1*, Δ*(rhaD-rhaB)568*, *hsdR514*). The parent of Keio collection is an *E. coli* strain BW25113 (F-, Δ*(araD-araB)567*, *ΔlacZ4787*(::rrnB-3), λ-, *rph-1*, Δ*(rhaD-rhaB)568*, *hsdR514*).

### Media

We used Milli-Q water for all media. Strains were grown with shaking (250 rpm) at 37 °C in LB medium (10 g of NaCl, 5 g of yeast extract and 10 g of tryptone per litre [l^−1^]) mixed with DM medium (0.5 g of C_6_H_5_Na_3_O_7_ · 2H_2_O, 1 g of (NH_4_)_2_SO_4_, 2 g of H_2_KO_4_P and 7 g of HK_2_O_4_P ·  3H_2_O l^−1^). 100 mg l^−1^ MgSO_4_ ·  7H_2_O (406 µmol) and 4 μg l^−1^ thiamine hydrochloride were added to DM after autoclaving. We used 1–90% LB with a content of Mg^2+^ ranging from 40 to 35.5 µmol, respectively, assuming that LB contains on average 35 µmol l^−1^ Mg^2+^ [35]. Selective tetrazolium arabinose agar (TA) medium (10 g of tryptone, 1 g of yeast extract, 5 g of NaCl, 3 g of arabinose and 0.05 g of 2,3,5-triphenyl-tetrazolium chloride l^−1^) was supplemented with freshly prepared rifampicin (50 µg ml^−1^) or nalidixic acid (30 µg ml^−1^). For all cell dilutions sterile saline (8.5 g l^−1^ NaCl) was used. All media were solidified as necessary with 15 g l^−1^ agar (Difco).

### Fluctuation tests

We conducted fluctuation tests with *E. coli* as already explained [6, 7]. In short, strains were first inoculated from frozen stock and grown in liquid LB medium at 37 °C and then transferred to nonselective liquid media (LB/DM) and allowed to grow overnight with shaking at 37 °C. *E. coli* cells were again diluted into fresh LB/DM, giving a mean initial population size (*N*_0_) of 2373 (range 1.5 × 10^2^–1.3 × 10^4^). Various volumes (0.35–1 ml) of parallel cultures were grown to saturation for 24 h at 37 °C in 96 deep-well plates. The position of each culture on a 96-deep-well polypropylene plate was chosen randomly. The final population size (*N*_*t*_) was determined by colony-forming units (CFU) where appropriate dilution was plated on solid nonselective TA medium. The final population density (*D*) estimated was determined by two independent techniques using CFU and an ATP-based assay: luminescence (LUM) was measured using a Promega GloMax luminometer and the Promega Bac-Titer Glo kit, according to the manufacturer’s instructions. We measured the luminescence of each culture 0.5 and 510 s after adding the Bac-Titer Glo reagent and calculated net luminescence as LUM = luminescence_510s_ − luminescence_0.5s_. Each estimate of *D* and *N*_*t*_ was averaged across three independent cultures. Evaporation (routinely monitored by weighing the plate before and after 24 h of incubation) was accounted for in the *N*_*t*_ value determined by CFU and was also used in statistical modelling as a variance covariate. We obtained the observed number of mutants resistant to rifampicin or nalidixic acid, *r*, by plating the entirety of the remaining cultures onto solid selective TA medium (4.5-cm plates in Figs. 1 and 2 and 9-cm plates in Fig. 3) that allows spontaneous mutants to form colonies. Plates were incubated at 37 °C, and mutants were counted at the earliest possible time after plating. For rifampicin plates, this was 44–48 h, when nalidixic acid was used the incubation time was 68–72 h.

For Figs. 1a, 2 and 3 we used 13, 3 and 4 independent experimental blocks, respectively, carried out on different days. Within an experimental block multiple 96-well plates were used. Any individual mutation rate estimate requires multiple parallel cultures, which were all carried out on a particular plate. For Figs. 1a, 2 and 3 the median (with an interquartile range) of parallel cultures used per mutation rate estimate was 16 (21–16), 16 (16–16) and 16 (16–16), respectively.

### Estimation of mutation rates

To estimate the number of mutational events, *m*, from the observed number of mutants, we employed the Ma-Sandri-Sarkar maximum-likelihood method implemented by the FALCOR web tool [36]. The mutation rate per cell per generation is calculated as *m* divided by the final population size, *N*_*t*_, determined by CFU. This approach does not account for potentially important issues that may affect mutation rate estimates. Crucially, if there is a cost to carrying a resistance allele in the fluctuation test environment, this can result in an underestimation of the mutation rate. This issue can be corrected for by co-estimating the average fitness effect of resistance mutations with the number of mutational events [37]. In addition, variation in *N*_*t*_ may also affect the estimates and may also be accounted for [38]. We therefore co-estimated mutation rates and fitness effects, accounting for variability in *N*_*t*_ using the flan package in R [39], also setting the Winsorization parameter to remove the effects of ‘jackpots’ with uncountably large numbers of mutants (greater than 150 on 4.5-cm plates and greater than 1000 on 9-cm plates). Since the estimated fitnesses (Fig. S9) tend to reinforce the patterns seen in Figs. 1–3, we report the results of the simpler and more widely used calculations in the main text as being more conservative.

### Statistical analysis

All statistical analysis was executed in R v3.3.1 [40] and nlme v3.1 packages for linear mixed effects modelling [41]. This enabled the inclusion within the same model of experimental factors (fixed effects), blocking effects (random effects) and factors affecting variance (giving heteroscedasticity), as described in Supplementary Information. In all cases log_2_ mutation rates were used. Details of models and their fitting are given in Supplementary Information: diagnostic plots in Supplementary Figures S13–S15, ANOVA tables for each model are given in Supplementary Tables S3–S5. The code and data to reproduce the main text figures are given in the accompanying R script, and Supplementary Data File, respectively. The content of the Supplementary Data File is explained in Supplementary Table S2.

## Electronic supplementary material

Supplementary Information

R script

Dataset 1
