# Supplementary material for: Opposing effects of final population density and stress on Escherichia coli mutation rate
Source: ISME J. 2018 Aug 7;12(12):2981–7. doi: 10.1038/s41396-018-0237-3 (PMC6230470; doi:10.1038/s41396-018-0237-3)
Supplement: Supplementary file 1 — Supplementary Information [file 41396_2018_237_MOESM1_ESM.pdf]

Supplementary Methods - Description of statistical models

1. Model S-I

The model of the data shown in Figure 1a fits $\log_2$ mutation rate against linear and quadratic effect of $\log_2$ percentage of lysogeny broth (%*LB*). For this and all subsequent models, an initial model was fit by restricted maximum likelihood (REML) including all fixed effects (in this case linear %*LB* and quadratic %*LB*) and all random effects (experimental plate nested within experimental block, each affecting the intercept). A series of model variants was then constructed allowing heteroscedasticity, i.e. an effect on variance of one or two covariates out of a range of features of individual mutation rate estimates which could affect the accuracy of that estimate. Diagnostic plots for this model are shown in Figure S13, further details are given in Table S3.

Potential variance covariates considered were discrete and continuous. Discrete were experimental block, strain, genotype identity and liquid growth media used. Continues were the fitted values of the response variable, the initial population size (N_0), the number of mutational events estimated (m), the coefficient of variation (CV) and standard deviation in that estimate, the initial LB concentration, the final population size (N_t) and its standard deviation, D (estimated by CFU or net luminescence (LUM) as available), net luminescence per cell (LUM/N_t), gross luminescence, absolute fitness, the number of generations, the generation time, phi ($1-N_t/N_0$), number and volume of parallel cultures used in the fluctuation test, proportion of weight remaining following evaporation during the growth of parallel cultures in the liquid media, upper bound, lower bound and range of the mutation rate estimate.

The model variant with the lowest AIC was then chosen. In this case the best model allowed variance to change with [fitted values of the mutation rate]$^{-4.7}$ x [lower bound of the mutation rate estimate]$^{2.3}$. No further simplification of the fixed effects was possible without significantly reducing the goodness of fit of the model. Here and below, a significant reduction in the goodness of fit was taken to mean $P < 0.05$ by likelihood ratio test, comparing models fit with and without particular fixed effects by maximum likelihood.

2. Model S-II

The model shown in Figure S5 (calibration curve) fits $\log_2$ final population density calculated with colony forming unts (D) against mean-centred $\log_2$ of luminescence (in arbitrary units from the ATP-based assay). It allows fixed effects of luminescence, genotype and their interaction and random effects of experimental plate nested within experimental block. The best model allowed variance to change with [fitted values of D]$^{-37.7}$ x [D]$^{1.9}$. No simplification of the fixed effects was possible without significantly reducing the goodness of fit of the model. This model was used to calibrate the luminescence values to give the final population densities used in Figures S4, S6 and S8. Diagnostic plots for this model are shown in Figure S14, further details are given in Table S4.

3. Model S-III

The model of the data shown in Figure 3 fits $\log_2$ mutation rate against linear and quadratic effect of $\log_2$ percentage of %*LB*. An initial model was fit by including all fixed

effects (in this case linear %*LB*, quadratic %*LB* and genotype and interactions genotype:linear %*LB* and genotype:quadratic %*LB*). Random effects of experimental plate nested within experimental block on the intercept were also included. The best model allowed variance to change with [fitted values of the mutation rate]$^{-10.8}$ x [upper bound of the mutation rate estimate]$^{3.8}$. No further simplification was possible without significantly reducing the goodness of fit of the model. Diagnostic plots for this model are shown in Figure S15, further details are given in Table S5.

Supplementary Figures

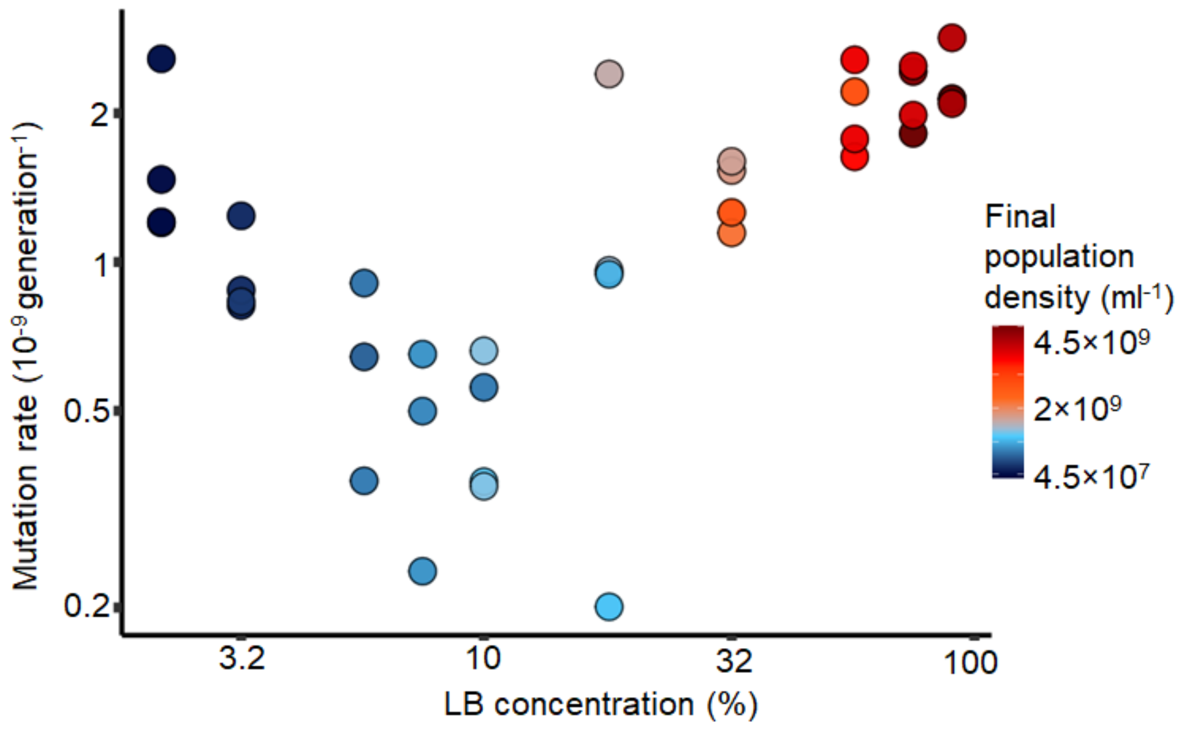

Figure S1. Effect of nutrient availability on mutation rate to nalidixic acid resistance in wild-type *E. coli* K-12 MG1655 (*N*=38). Cells were grown in Davis minimal medium mixed with 1% to 90% of lysogeny broth (LB) medium. Colours represent final population density measured by colony forming units (see Fig. S2 for details). Note the non-linear axes.

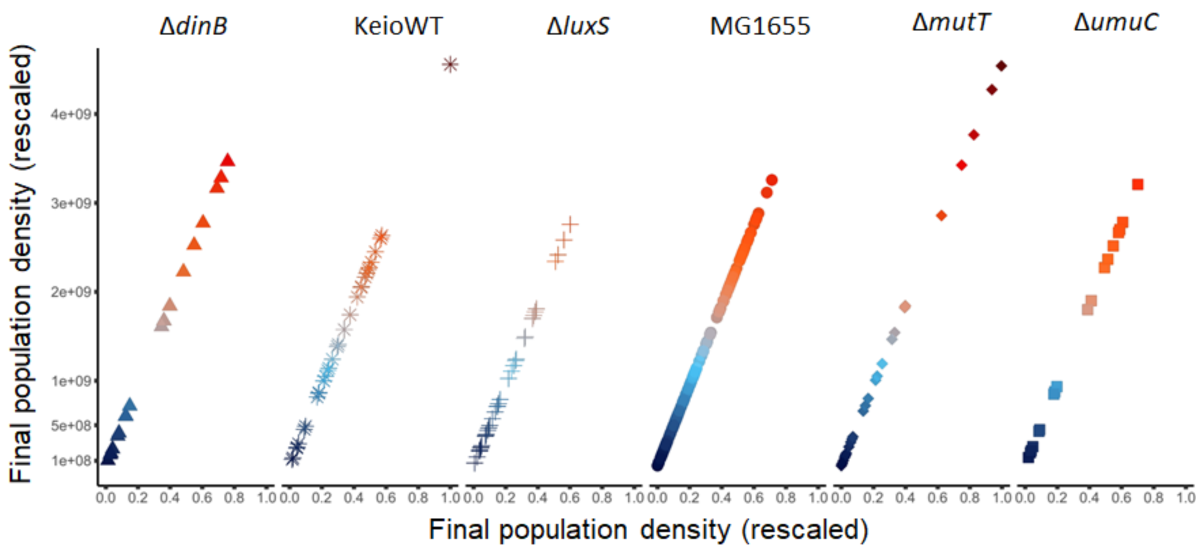

Figure S2. Final population density measured by colony forming units (CFU) of six *E. coli* strains used in this study (*N*=271). This colour scheme is used in Figures 1, 2, 3, S1, S3 and S10-S12.

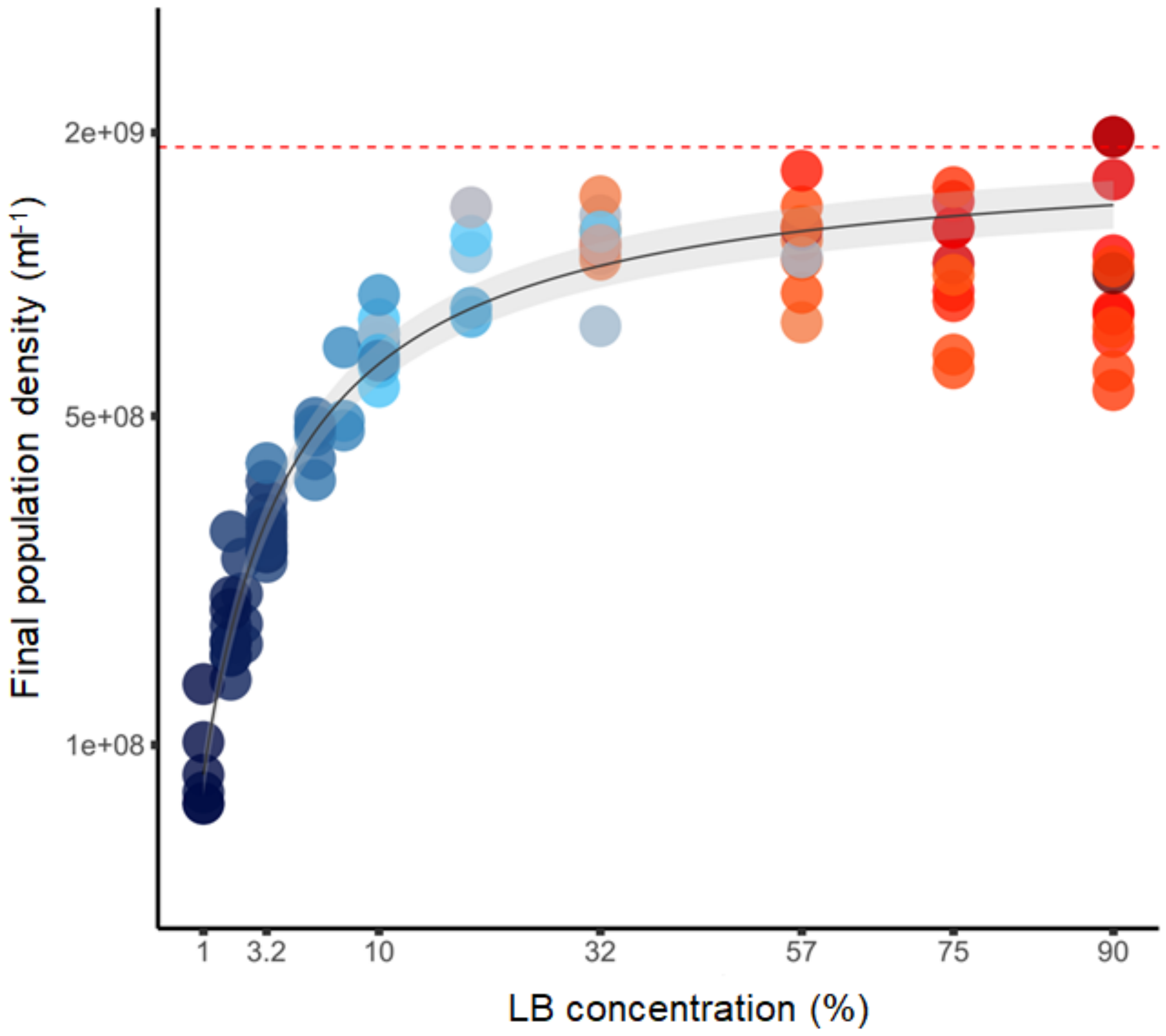

Figure S3. Final population density against percentage of lysogeny broth medium in *E. coli* MG1655. Red line is maximum final population density predicted by the model (*N*=97). Final population density is measured by ATP assay (see Figure S5), see also Methods. Note the non-linear axes.

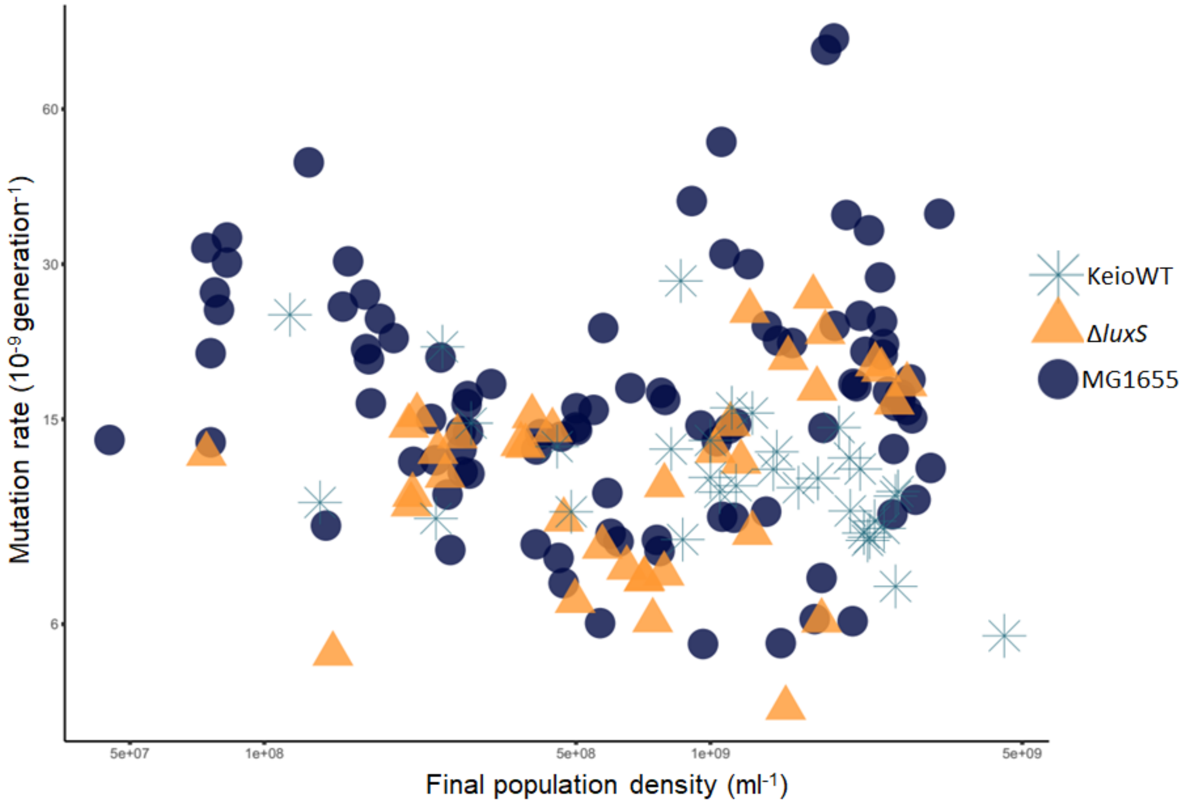

Figure S4. Effect of final population density on mutation rate to rifampicin resistance in three genotypes: *E. coli* MG1655 wild-type (dark blue circles, *N*=97), Δ*luxS* (orange triangles, *N*=37) and Keio parent (turquoise snowflakes, *N*=33). Cells were grown in Davis minimal medium mixed with 1% to 90% of lysogeny broth (LB) medium. Final population density was measured with colony forming units. Note the non-linear axes.

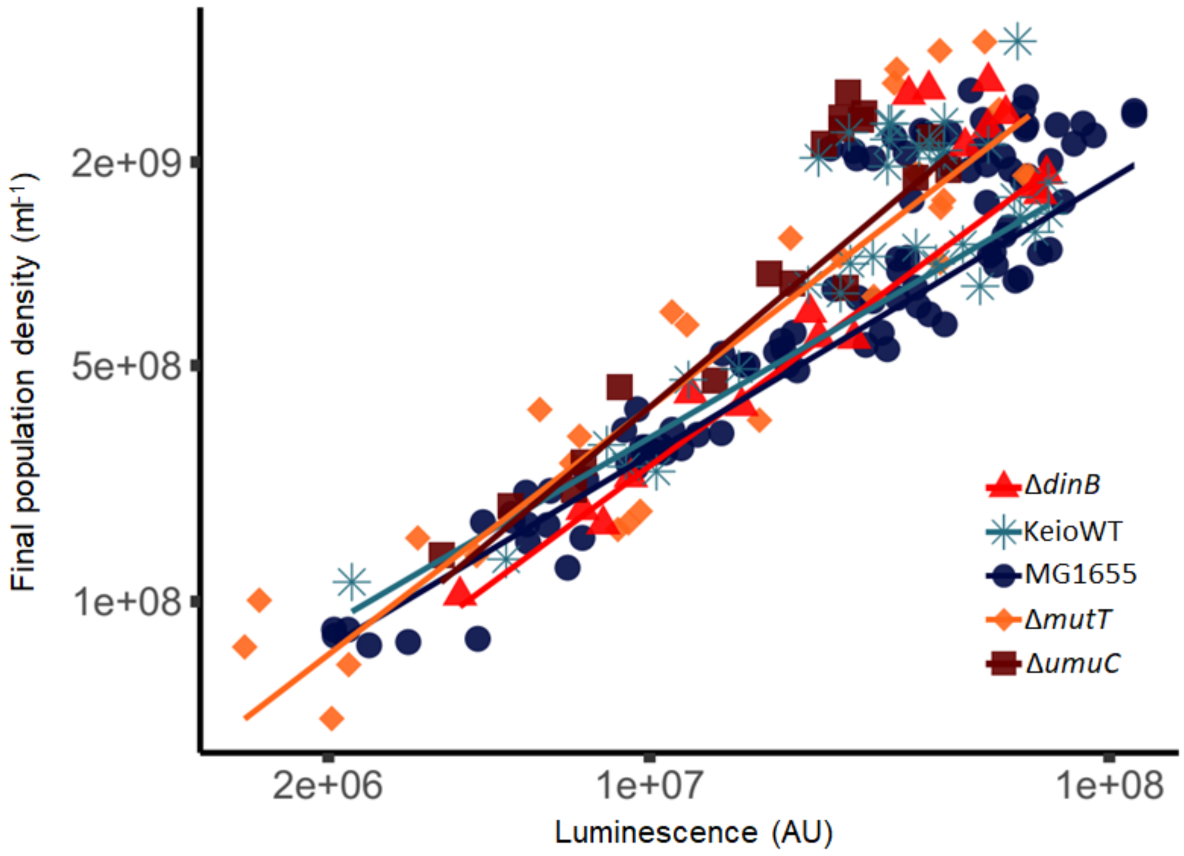

Figure S5. Calibration curve for final population density measured by counting colony forming units (CFU) against luminescence using an ATP-based assay (arbitrary units). Calibration curves are for *E. coli* MG1655 (dark blue circles) and for four Keio strains Δ*dinB* (red triangles), Δ*mutT* (orange diamonds), Δ*umuC* (brown squares) and their parent (turquoise snowflakes). Lines are from a Model S-II (*N*=196) that was used in Figures S3, S6 and S8. See Methods for further strain details.

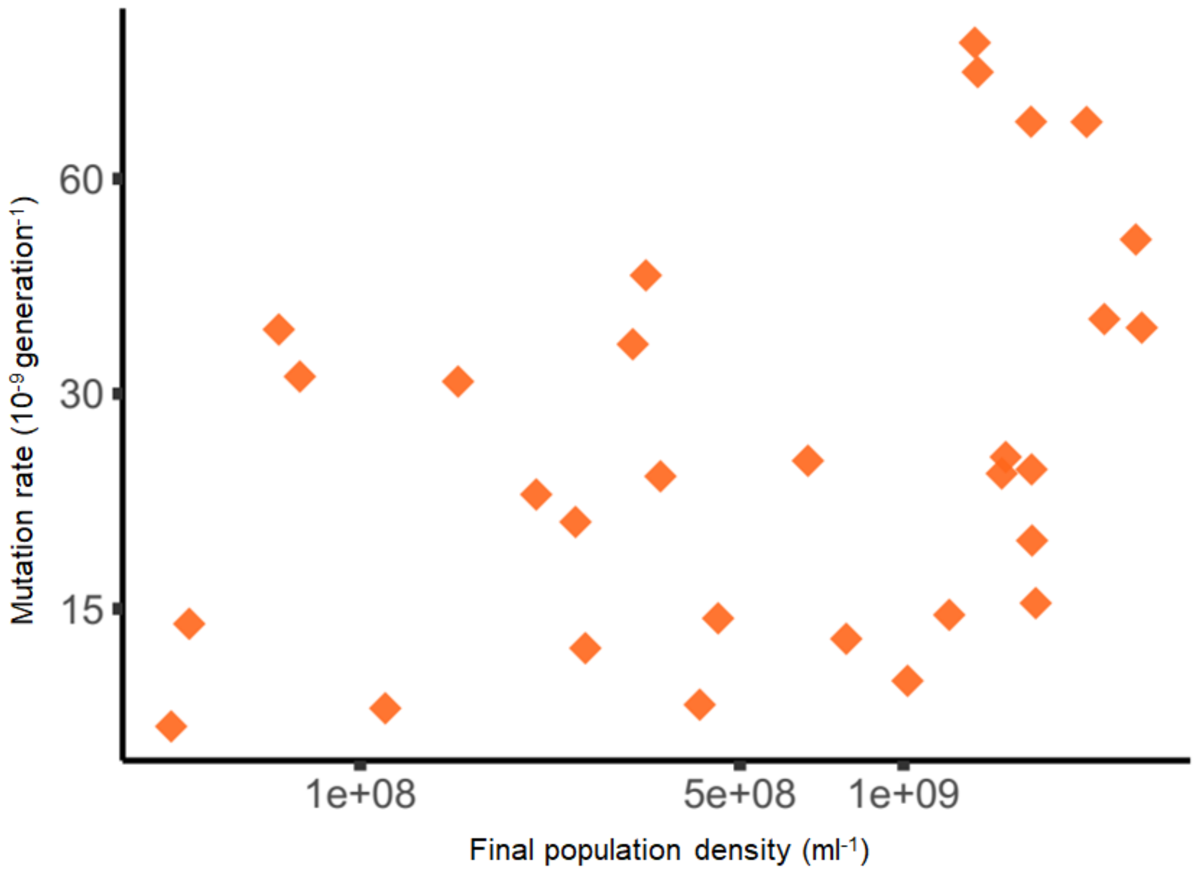

Figure S6. Effect of final population density on mutation rate to nalidixic acid resistance in *E. coli* Δ*mutT* (*N*=30). Cells were grown in Davis minimal medium mixed with 1% to 90% of lysogeny broth (LB) medium. Final population density was measured with ATP-based luminescence assay (see Figure S5 and Methods). Note the non-linear axes.

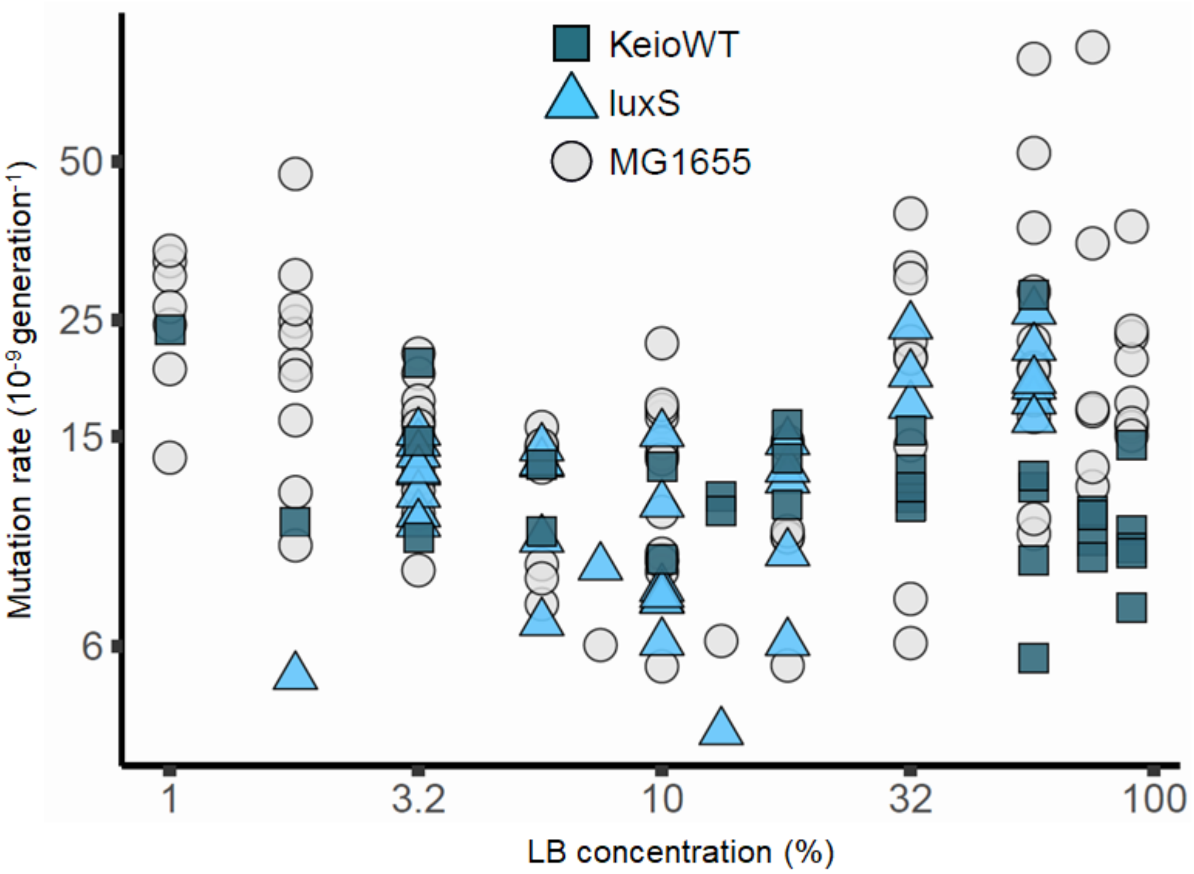

Figure S7. Effect of nutrient availability on mutation rate to rifampicin resistance in three genotypes: *E. coli* MG1655 wild-type (light grey circles, *N*=97), Δ*luxS* (light blue triangles, *N*=37) and Keio parent (turquoise squares, *N*=33). Cells were grown in Davis minimal medium mixed with 1% to 90% of lysogeny broth (LB) medium. Note the non-linear axes.

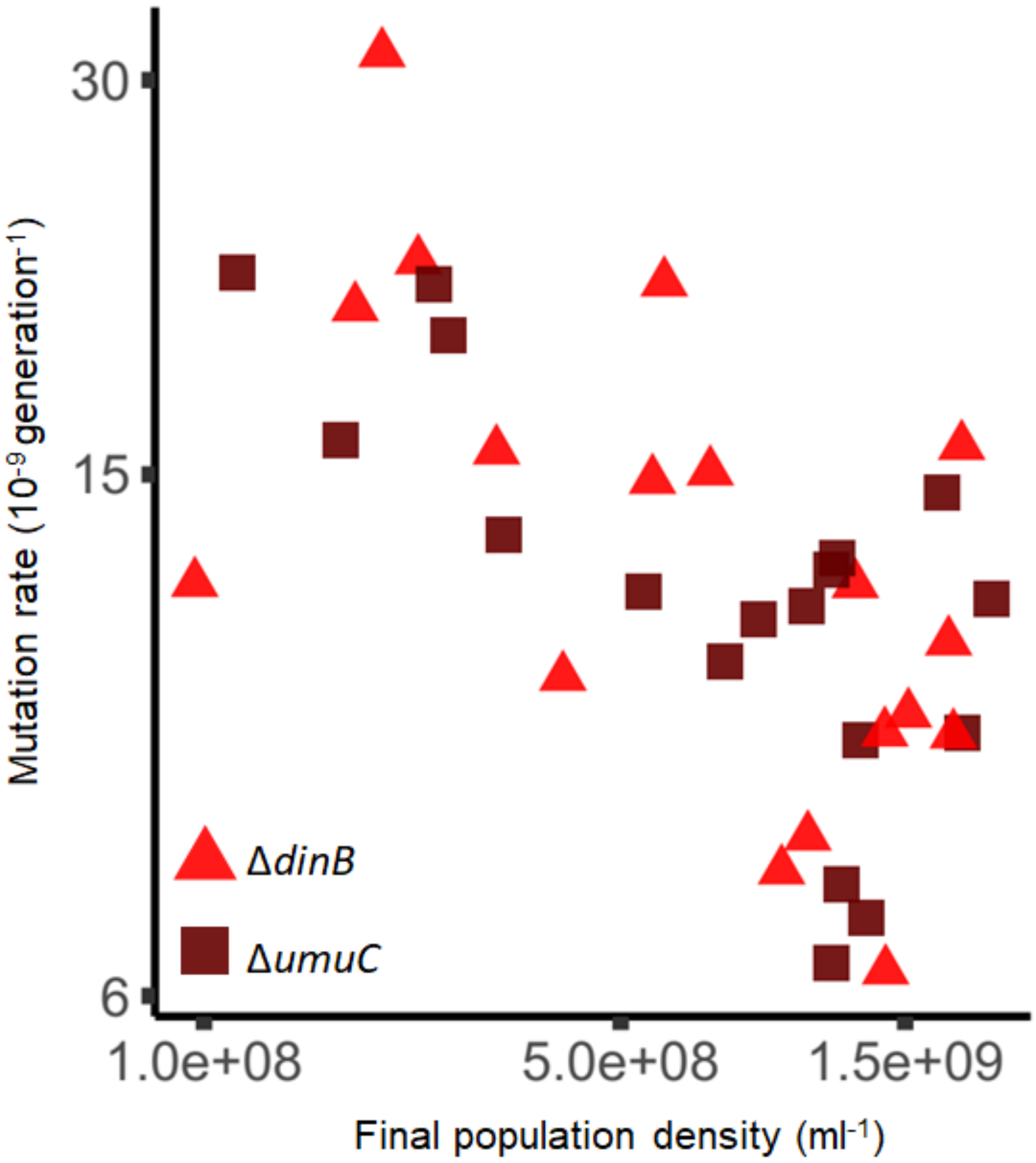

Figure S8. Effect of final population density on mutation rates to rifampicin resistance in cells without error-prone polymerases Δ*dinB* (*N*=18, triangles) and Δ*umuC* (*N*=18, squares). Distribution of mutation rates among both genotypes is indistinguishable across population densities (*N*=36). Cells were grown in Davis minimal medium mixed with 1% to 90% of lysogeny broth (LB) medium. Final population density was measured with ATP-based luminescence assay (see Figure S5 and Methods). Note the non-linear axes.

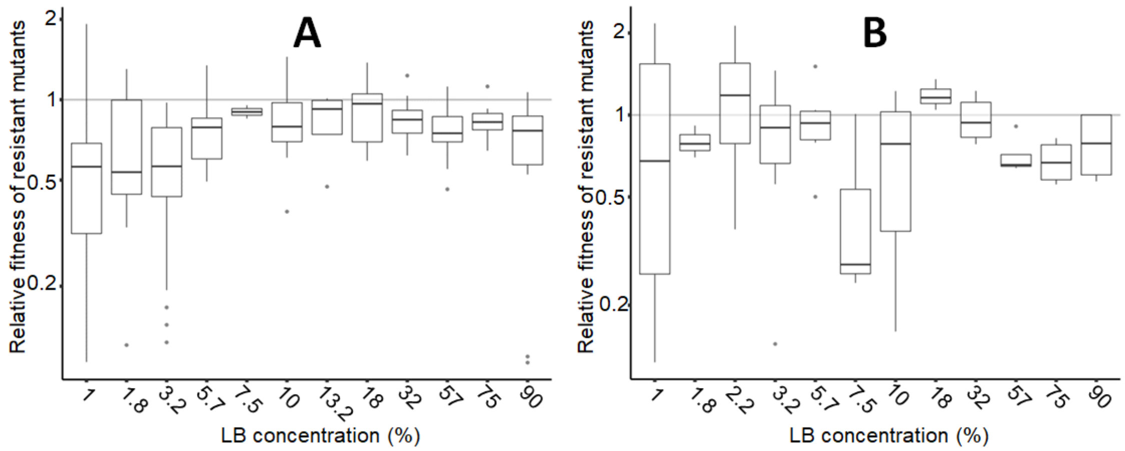

Figure S9. Average fitness effects of resistance mutations to **A**) rifampicin (*N*=203) and **B**) nalidixic acid (*N*=68), co-estimated with mutation rate (see Methods). Box plots show the distributions of the best estimate of the average relative fitness of a resistant mutant in each fluctuation test. Bars indicate medians, boxes indicate inter-quartile ranges (25th-75th percentiles), whiskers extend to data points no more than1.5 times the interquartile range from the edge of the box and points indicate any data points falling beyond the whisker. The co-estimated mutation rates are shown in Figs. S10 and S12 (**A**) and Fig. S11 (**B**). Note the non-linear axes.

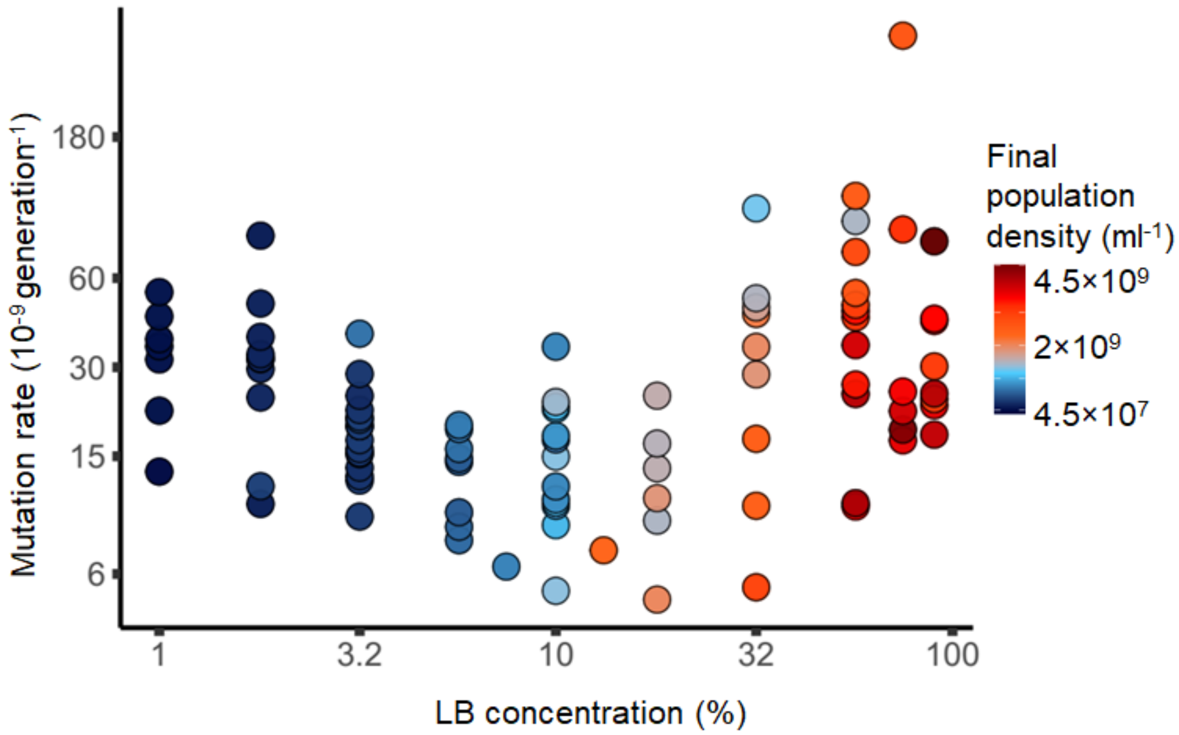

Figure S10. Effect of nutrient availability on mutation rate to rifampicin resistance in wild-type *E. coli* K-12 MG1655 (*N*=97). Unlike Fig. 1, mutation rates here were co-estimated with the relative fitness of resistant mutants (Fig. S9), while accounting for variability in *Nt* (see Methods). Cells were grown in Davis minimal medium mixed with 1% to 90% of lysogeny broth (LB) medium. Colours represent final population density measured by colony forming units (see Fig. S2 for details). Note the non-linear axes.

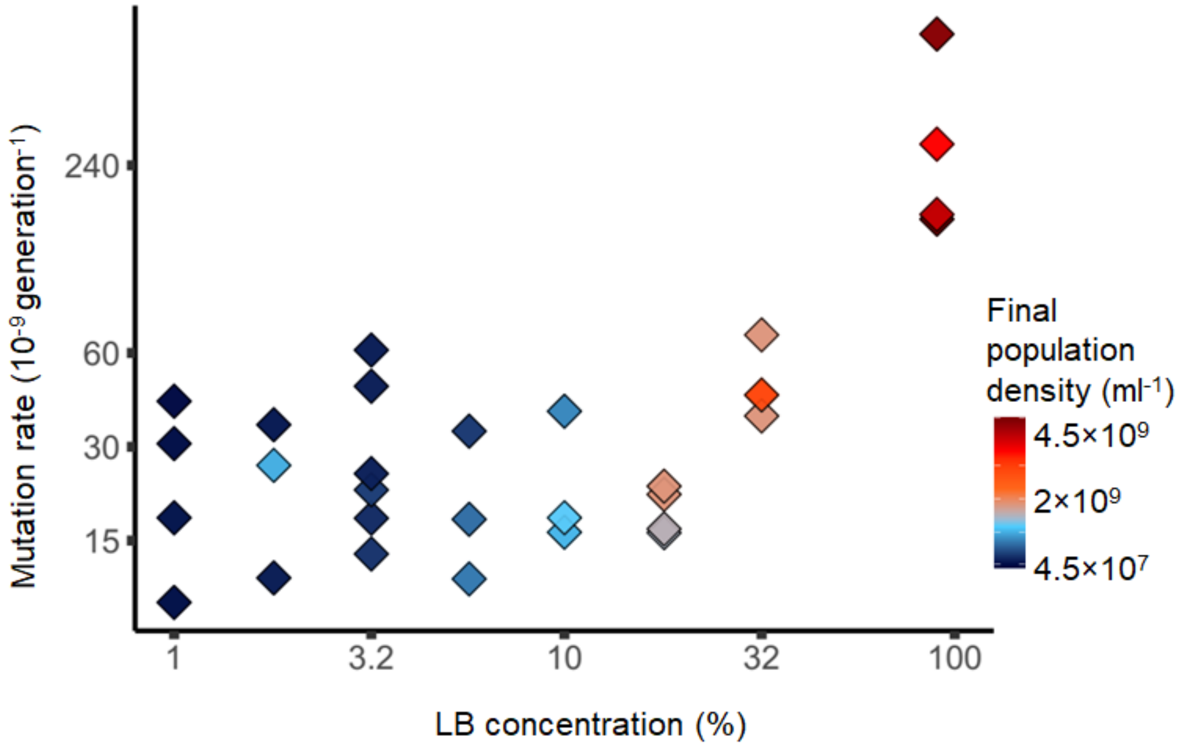

Figure S11. Effect of nutrient availability on mutation rate to nalidixic acid resistance in cells without DAMP (Δ*mutT*, *N*=30). Unlike Fig. 2, mutation rates here were co-estimated with fitness effects (see Fig. S9), while accounting for variability in *Nt* (see Methods). Cells were grown in Davis minimal medium mixed with 1% to 90% of lysogeny broth (LB) medium. Colours represent final population density measured by colony forming units (see Fig. S2 for details). Note the non-linear axes.

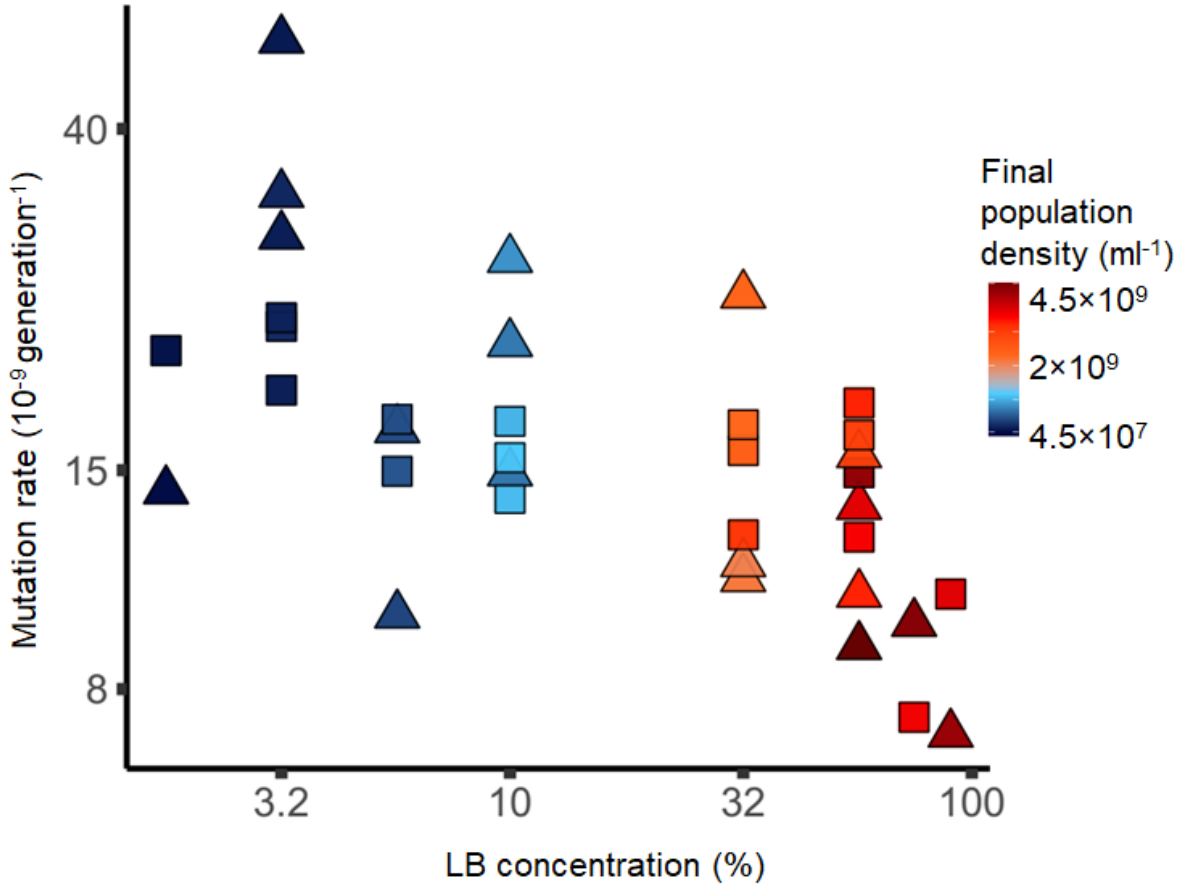

Figure S12. Effect of nutrient availability on mutation rates to rifampicin resistance in cells without error-prone polymerases Pol IV (Δ*dinB*, *N*=18, triangles) and Pol V (Δ*umuC*, *N*=18, squares). Unlike Fig. 3, mutation rates here were co-estimated with fitness effects (see Fig. S9), while accounting for variability in *Nt* (see Methods). Cells were grown in Davis minimal medium mixed with 1% to 90% of lysogeny broth (LB) medium. Colours represent final population density measured by colony forming units (see Fig. S2 for details). Note the non-linear axes.

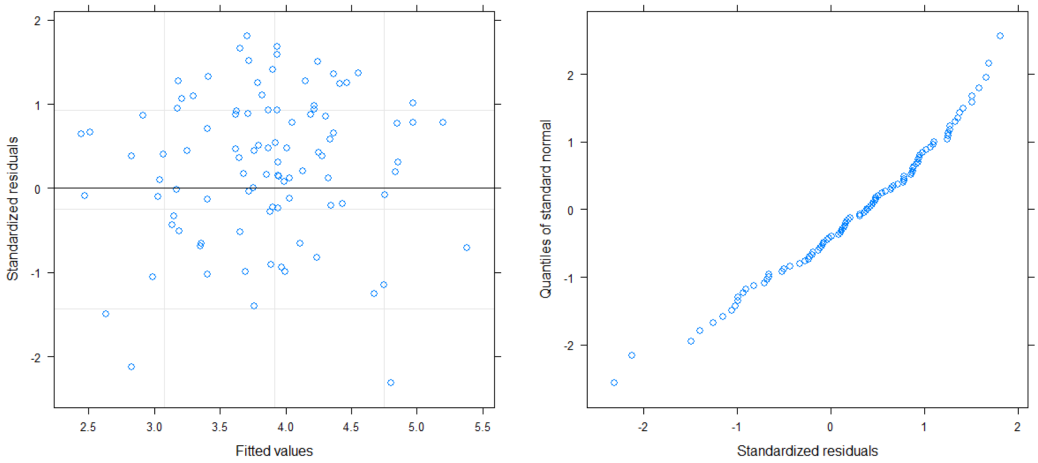

Figure S13. Diagnostic plots for Model S-I (Figure 1a). Standardised residuals by fitted values and normal quantile-quantile plot of standardised residuals. Further details are given in Table S3.

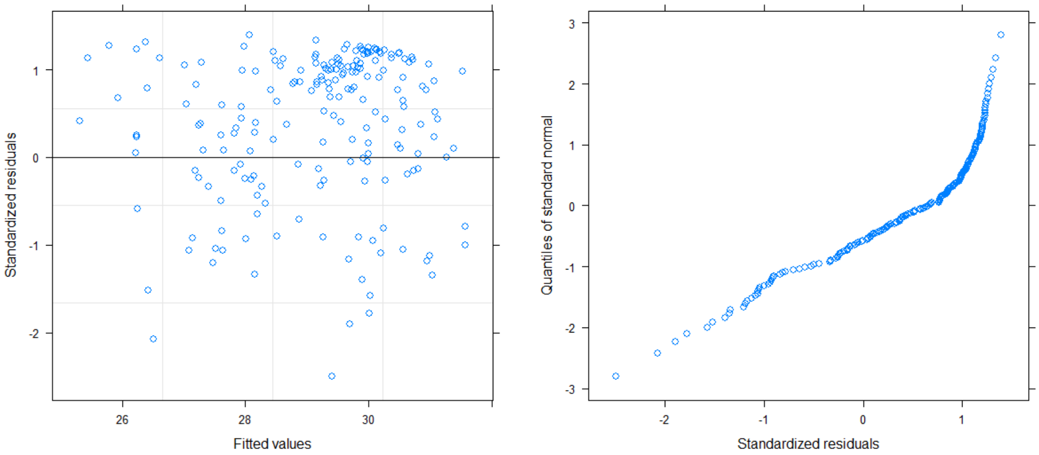

Figure S14. Diagnostic plots for Model S-II (Figure S5). Standardised residuals by fitted values and normal quantile-quantile plot of standardised residuals. Further details are given in Table S4.

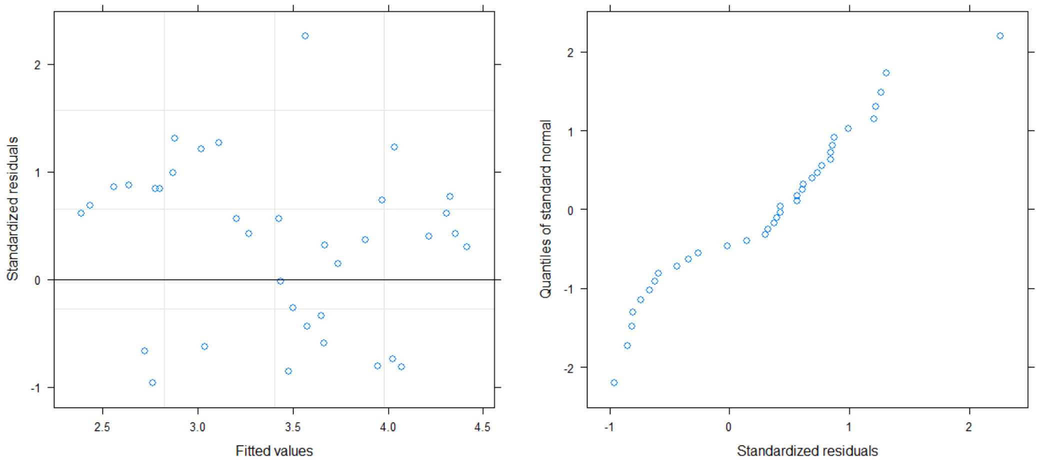

Figure S15. Diagnostic plots for Model S-III (Figure 3). Standardised residuals by fitted values and normal quantile-quantile plot of standardised residuals. Further details are given in Table S5.

Supplementary Tables

Table S1. List of papers used in Figure 4.

1.	C. Aguilar *et al.*, Deletion of the 2-acyl-glycerophosphoethanolamine cycle improve glucose metabolism in *Escherichia coli* strains employed for overproduction of aromatic compounds. *Microb. Cell. Fact.* **14**, 194 (2015).
2.	F. I. Arias-Sánchez, A. R. Hall, Effects of antibiotic resistance alleles on bacterial evolutionary responses to viral parasites. *Biol. Lett.* **12**, 20160064 (2016).
3.	B. Csörgo, T. Fehér, E. Tímár, F. R. Blattner, G. Pósfai, Low-mutation-rate, reduced-genome *Escherichia coli*: an improved host for faithful maintenance of engineered genetic constructs. *Microb. Cell. Fact.* **11**, 11 (2012).
4.	M. Demerec, Studies of the Streptomycin-Resistance System of Mutations in *E. Coli. Genetics* **36**, 585-597 (1951).
5.	M. Demerec, U. Fano, Bacteriophage-Resistant Mutants in *Escherichia coli*. *Genetics* **30**, 119 (1945).
6.	S. M. Karve *et al.*, *Escherichia coli* populations in unpredictably fluctuating environments evolve to face novel stresses through enhanced efflux activity. *J. Evol. Biol.* **28**, 1131-1143 (2015).
7.	P. Komp Lindgren, Å. Karlsson, D. Hughes, Mutation Rate and Evolution of Fluoroquinolone Resistance in *Escherichia coli* Isolates from Patients with Urinary Tract Infections. *Antimicrob. Agents Chemother*. **47**, 3222-3232 (2003).
8.	S. E. Luria, M. Delbrück, Mutations of bacteria from virus sensitivity to virus resistance. *Genetics* **28**, 491-511 (1943).
9.	H. B. Newcombe, Delayed Phenotypic Expression of Spontaneous Mutations in *Escherichia Coli*. *Genetics* **33**, 447-476 (1948).
10.	H. B. Newcombe, R. Hawirko, Spontaneous Mutation to Streptomycin Resistance and Dependence in *Escherichia coli*. *J. Bacteriol.* **57**, 565-572 (1949).
11.	H. B. Newcombe, G. J. Mc, On the nonadaptive nature of change to full streptomycin resistance in *Escherichia coli*. *J. Bacteriol.* **62**, 539-544 (1951).
12.	C. Riesenfeld, M. Everett, L. J. V. Piddock, B. G. Hall, Adaptive mutations produce resistance to ciprofloxacin. *Antimicrob. Agents Chemother.* **41**, 2059-2060 (1997).

Table S2. Detailed description of the columns in the raw data file Krasovec_etal_data_ISME.csv

"marker"	Phenotypic (selective) marker used in the fluctuation test.
"figure"	The figure in which the row of data is used.
"mutation_rate"	The estimated mutation rate per genome per generation multiplied by 10^9.
"percentage_of_LB"	The percentage of lysogeny broth in "environment".
"genotype"	The genotype of the strain in which mutation rate was measured (luxS, MG1655, KeioWT, mutT, dinB and umuC, corresponding to *Escherichia coli* K-12 strain KX1228 *(ΔluxS)*, *wild-type* K-12 MG1655, *E. coli* Keio parent BW25113 and JW0097-1 *(ΔmutT)*, JW0221-1 (Δ*dinB*) and JW1173-1 (Δ*umuC*) from Keio collection, respectively. See Methods for more details.
"Authors"	List of authors of the paper containing mutation rates used in Figure 4. For the entire reference see Table S1.
"culture_volume"	The initial volume of parallel cultures.
"N0"	The initial population size of cells in parallel cultures.
"Nt"	The population size at the end of the culture period estimated via colony forming units averaged over three parallel cultures.
"D"	The estimated number of cells per ml at the end of the culture period calculated with colony forming units and averaged over three parallel cultures.
"LUM"	Net luminescence (LUM_{510}-$LUM_{0.5}$) in arbitrary units measured with luminometer using ATP-based assay.
"D2"	Scaled "D" used in Figure S2.
"culture_1"	Number of observed mutants in a parallel culture no. 1.
"culture_2"	Number of observed mutants in a parallel culture no. 2.
"culture._3"	Number of observed mutants in a parallel culture no. 3.
"culture_4"	Number of observed mutants in a parallel culture no. 4.
"culture_5"	Number of observed mutants in a parallel culture no. 5.
"culture_6"	Number of observed mutants in a parallel culture no. 6.
"culture_7"	Number of observed mutants in a parallel culture no. 7.
"culture_8"	Number of observed mutants in a parallel culture no. 8.
"culture_9"	Number of observed mutants in a parallel culture no. 9.
"culture_10"	Number of observed mutants in a parallel culture no. 10.
"culture_11"	Number of observed mutants in a parallel culture no. 11.
"culture_12"	Number of observed mutants in a parallel culture no. 12.
"culture_13"	Number of observed mutants in a parallel culture no. 13.
"culture_14"	Number of observed mutants in a parallel culture no. 14.
"culture_15"	Number of observed mutants in a parallel culture no. 15.
"culture_16"	Number of observed mutants in a parallel culture no. 16.
"culture_17"	Number of observed mutants in a parallel culture no. 17.

"culture_18"	Number of observed mutants in a parallel culture no. 18.
"culture_19"	Number of observed mutants in a parallel culture no. 19.
"culture_20"	Number of observed mutants in a parallel culture no. 20.
"culture_21"	Number of observed mutants in a parallel culture no. 21.

Table S3. ANOVA Table and fitted values for Model S-I (Figure 1a).

See Materials and Methods and Figure S13 for more details.

	Degrees of freedom	Value	SE	*F*	*P*
Intercept	1	4.2	0.17	747	4.3×10^{-33}
$\log_2(LB)$	1	-0.98	0.084	108	6.1×10^{-17}
$(\log_2[LB])^2$	1	0.25	0.016	240	1.8×10^{-22}

Table S4. ANOVA Table and fitted values for Model S-II (Figure S5).

See Materials and Methods and Figure S14 for more details.

	Degrees of freedom	Value	SE	*F*	*P*
Intercept (Δ*dam*)	1	29	0.19	80561	9.9×10^{-163}
$\log_2(\text{LUM})_{centred}$	1	1.0	0.048	3468	8.9×10^{-46}
genotype (Keio parent)	4	0.032	0.12	8.6	
(MG1655)		-0.11	0.24		
(Δ*mutT*)		0.62	0.31		
(Δ*umuC*)		0.74	0.12		
$\log_2(\text{LUM})_{centred}$:(Keio parent)	4	-0.21	0.066	15	
$\log_2(\text{LUM})_{centred}$:(MG1655)		-0.19	0.052		
$\log_2(\text{LUM})_{centred}$:(Δ*mutT*)		0.039	0.059		
$\log_2(\text{LUM})_{centred}$:(Δ*umuC*)		0.14	0.078		

Table S5. ANOVA Table and fitted values for Model S-III (Figure 3).

See Materials and Methods and Figure S15 for more details.

	Degrees of freedom	Value	SE	*F*	*P*
Intercept (Δ*dinB*)	1	3.8	0.27	864	7.1×10^{-13}
$\log_2(LB)$	1	0.30	0.18	376	0.11
$(\log_2[LB])^2$	1	-0.11	0.030	0.0080	0.0016
genotype	1	1.1	0.33	2.4	0.043
$\log_2(LB)$:genotype (Δ*umuC*)	1	-0.98	0.23	18	0.00026
$(\log_2[LB])^2$:genotype Δ*umuC*	1	0.14	0.041	12	0.0018
